# Public Perception of the Tobacco 21 Amendment on Twitter in the United States: Observational Study

**DOI:** 10.2196/53899

**Published:** 2024-09-25

**Authors:** Liane M Schneller-Najm, Zidian Xie, Jiarui Chen, Sarah Lee, Emily Xu, Dongmei Li

**Affiliations:** 1 Department of Health Behavior Roswell Park Comprehensive Cancer Center Buffalo, NY United States; 2 Department of Clinical and Translational Research University of Rochester Medical Center Rochester, NY United States; 3 Goergen Institute for Data Science University of Rochester Rochester, NY United States

**Keywords:** tobacco policy, tobacco regulation, social media, tobacco use, tobacco, health belief, sentiment analysis, smoking, cigarettes, social media analysis, vaping, e-cigarettes, health behavior, public opinion

## Abstract

**Background:**

Following the signing of the Tobacco 21 Amendment (T21) in December 2019 to raise the minimum legal age for the sale of tobacco products from 18 to 21 years in the United States, there is a need to monitor public responses and potential unintended consequences. Social media platforms, such as Twitter (subsequently rebranded as X), can provide rich data on public perceptions.

**Objective:**

This study contributes to the literature using Twitter data to assess the knowledge and beliefs of T21.

**Methods:**

Twitter data were collected from November 2019 to February 2021 using the Twitter streaming application programming interface with keywords related to vaping or e-cigarettes, such as “vape,” “ecig,” etc. The temporal trend of the T21 discussion on Twitter was examined using the mean number of daily T21-related tweets. Inductive methods were used to manually code the tweets into different sentiment groups (positive, neutral, and negative) based on the attitude expressed toward the policy by 3 coders with high interrater reliability. Topics discussed were examined within each sentiment group through theme analyses.

**Results:**

Among the collected 3197 tweets, 2169 tweets were related to T21, of which 444 tweets (20.5%) showed a positive attitude, 736 (33.9%) showed a negative attitude, and 989 (45.6%) showed a neutral attitude. The temporal trend showed a clear peak in the number of tweets around January 2020, following the enactment of this legislation. For positive tweets, the most frequent topics were “avoidance of further regulation” (120/444, 27%), “Enforce T21” (110/444, 24.8%), and “health benefits” (81/444, 18.2%). For negative tweets, the most frequent topics were “general disagreement or frustration” (207/736, 28.1%) and “will still use tobacco” (188/736, 25.5%). Neutral tweets were primarily “public service announcements (PSA) or news posts” (782/989, 79.1%).

**Conclusions:**

Overall, we find that one-third of tweets displayed a negative attitude toward T21 during the study period. Many were frustrated with T21 and reported that underage consumers could still obtain products. Social media data provide a timely opportunity to monitor public perceptions and responses to regulatory actions. Continued monitoring can inform enforcement efforts and potential unintended consequences of T21.

## Introduction

Nearly all current daily adult cigarette users reported first trying a cigarette before the age of 18 years. While cigarette use has decreased among adults, as well as youth, other products, such as electronic nicotine delivery systems, have gained popularity. In 2023, 12.6% of high schoolers reported current use of tobacco (in the past 30 days), including cigarettes, cigars, smokeless tobacco, hookah, heated tobacco products, and electronic nicotine delivery systems [1]. Furthermore, 3.9% of high schoolers reported current use of 2 or more products in the past 30 days [1] and are therefore more likely to continue nicotine use into adulthood [2-4]. In order to prevent tobacco initiation, the Tobacco 21 Amendment (T21) of the Federal Food, Drug, and Cosmetic Act was signed in December 2019 [5]. T21 raised the federal minimum age to purchase tobacco products from 18 to 21 years [5].

T21 was vital to interrupting the US youth and early young adult tobacco epidemic as tobacco product use is the leading cause of preventable diseases and death [2], and most current tobacco users started by the age of 18 [2,3]. Youth may obtain tobacco products from social sources, including peers and classmates, as well as commercial sources [6,7]. Before the Federal T21 legislation, 19 states and many more localities passed legislation to increase the minimum age of tobacco sales as early as January 2016 [8]. Simulations and many other evaluations of local legislation found that raising the minimum age to purchase tobacco reduced product sales [9-11], product use prevalence [12-20], initiation of nicotine use [13,21], and tobacco-related health disparities among youth [20]. Therefore, one would expect to see similar changes at a national level. However, underage tobacco sales and use have been an issue for decades and require retailer compliance, with more youth and early young adults reporting purchasing their products directly from the retailer than any other source [6,7]. In April 2018, the Truth Initiative surveyed 12- to 17-year-old, past 30-day JUUL (a vape system developed by Juul Labs, Inc) users nationally and found that 74% (n/N) of them purchased their product from a retailer, 52% (n/N) from a social source such as a friend or family member, and 6% (n/N) web-based retailers [6]. Therefore, it is important to prevent youth smokers from acting as suppliers and to make it more difficult for 16- and 17-year-olds to pass as legal purchasers. Policy enforcement and retailer compliance are crucial to reducing tobacco use among youth [11,22]. Furthermore, social determinants have been shown to impact retailer compliance, including previous state and local tobacco control policies, neighborhood demographics, retail signage, and scanners for ID checks [23-25]. A study assessing the impact of the Federal T21 after 1 year of signing found that middle- and high-school students perceived it to be more difficult to buy tobacco products from a store, but this was not the case for purchasing products from web-based retailers [25].

Following T21, there is an urgent need to monitor the knowledge and beliefs of T21. Before the Federal T21 policy, previous studies have assessed attitudes toward raising the minimum age to purchase tobacco products. In general, the studies found that the majority of adults were in favor of increasing the age to purchase tobacco to 21 years, regardless of smoking status and demographics [26-28]. Social media platforms, such as Twitter (subsequently rebranded as X), can provide rich data on public perceptions. In the past, Twitter has been used to examine discussions focused on government policies, such as e-cigarette flavor policies and T21 [29-34]. Many tweets in these previous studies that used Twitter data to assess sentiment toward T21 identified a neutral tone toward T21; however, an alternative to previous survey findings, more tweets (over one-third) portrayed opposition to the policy rather than support [32-34]. Common themes identified in these studies included unfairness to youth who were already addicted to nicotine and skepticism toward the policy efficacy [32], a disjunction for other age restrictions (eg, military, alcohol, and voting) [33], and incorrectly describing the policy as a purchase law [34]. However, these Twitter studies used data from the months leading up to the signing of the amendment. This study uses Twitter data a month before and over a year following the signing of the amendment to assess the public attitude toward T21 and its potential impact on tobacco use behavior, such as policy avoidance or seeking out cessation advice.

## Methods

### Data Collection

Publicly available Twitter data from November 12, 2019, to February 26, 2021, was previously collected using the Twitter streaming application programming interface with keywords related to vaping or e-cigarettes for a previous study [35]. The methodology and vaping or e-cigarette keywords can be found published elsewhere [35]. Next, we filtered out a subset of the data using keywords related to the T21 policy, such as “tobacco21,” “tobacco 21,” “t21,” “tobacco age,” “vaping age,” “tobacco purchase law,” “buy tobacco,” “tobacco age restriction,” “tobacco 21 laws”, “minimum age to buy tobacco,” “vape age,” “smoking age,” “legal age,” and “tobacco age.” The tweets without any of the keywords were eliminated. Then, we applied 2 filters to remove tweets related to the commercial promotion of smoking and vaping products [35]. The first filter was applied to the Twitter username, and the keyword list contained “dealer,” “store,” “promo,” etc. If a tweet is associated with a username containing any of these keywords, it is removed from the data set. The second filter targeted the tweet content. The keywords contained, but were not limited to, “discount,” “sale,” “percent off,” and “store.” Finally, repetitive tweets and retweets were removed, and we obtained a data set with 6489 tweets. In addition, any additional commercial tweets were manually excluded from the data set, resulting in 49.3% (3197/6489) of tweets being hand-coded. 

### Content Analysis

Content analyses were conducted from April 2022 to May 2022 using inductive methods. From the processed data set, we randomly selected 10% (320/3197) of the tweets to manually review the tweets, identify key attitudes and themes, and develop the codebook for the content analysis of the entire data set. First, we determined whether each tweet was related to the T21 policy, and then we grouped the related tweets into 3 categories (positive, negative, and neutral) based on the attitude expressed toward the policy. Next, we identified the topics of supportive and antagonistic reasoning and labeled each positive and negative tweet with the identified topic. Neutral tweets were labeled in a slightly different way due to the lack of apparent reasoning and were divided into “public service announcements (PSA) or news,” “dialogue or discussion,” and “extend to other regulations.” A list of all topics and their definitions are shown in Table 1.

When developing the codebook, 2 coders reviewed the tweet independently and respectively summarized the topics. Then the commons and differences between the 2 versions were collectively discussed among all 6 authors to reach a consensus on the final codebook. Next, the 2 coders reviewed sample tweets again to adjust their original results according to the revised codebook. The 2 coders achieved an overall kappa agreement of 0.80 on attitudes and an overall rate of 0.74 on the specific topic labels. For the disagreement, a third coder was introduced to resolve the discrepancy during the development of the codebook. After the codebook was developed, the remaining tweets were divided into 3 parts and were labeled by 3 coders separately. Each tweet was identified into 1 attitude group and then one of the topics within the attitude group. We chose not to collectively label all the remaining tweets due to the large number of tweets. Finally, the results for sample tweets and the remaining tweets were combined for analysis.

**Table 1:** Codebook for hand-coding Tobacco 21–related tweets.

Attitude and topic		Description	Examples
**Positive**			
	Avoidance of further regulation	The tweet claims that the governors should let T21^a^ do its job before implementing additional regulations.	“as an adult, I will not have my rights stripped away, including flavors. Tobacco 21 will be the best solution. we have respiratory therapists who recommend vaping over smoking, no use of thc!”
	Enforce T21	The tweet calls for thorough execution and enforcement of the policy.	“it's already illegal for children to buy tobacco and vaping products. how about this: how about you release who funds your little group? and yes, protects the childrens. protect them by enforcing the laws already on the books. stop the hysteria!”
	Health benefit	The tweet states that T21 reduces the harm to the health of the youth.	“congress should act to ban characterizing flavors in all tobacco products, give fda user fees to regulate e-cigs, and raise tobacco age to 21. these steps can combat youth tobacco use, preserve harm reduction, and further reduce tobacco death and disease.”
	General support	The tweet offers no specific reason for supporting T21 but generally expresses a positive attitude.	“tobacco 21 legislation is critical! learn more about youth tobacco use in the granite state here.”
	Reduce usage	The tweet states that T21 will be effective in reducing the usage of tobacco in the youth.	“for me flavours play a critical role in disassociating vaping from smoking. lots of others feel the same, it's a matter of making the product better than smoking, and thus more enjoyable than smokes. t21 keeps the product out of the hands of kids.”
	Other	Other tweets with positive attitudes that do not belong to any of the other categories.	“wow, believe your listening to us vapers! children should not vape or smoke, educating parents is very important! t21 is the law and should be followed! if i had this when i was 16, i would of never started smoking! #vaping is for adults who want to quit smoking!”
**Negative**			
	Parenting or blaming	The tweet argues that the essence of the problem is not tobacco, but other factors such as advertisement, parenting, etc.	“i agree that the shops should be held accountable and that t-21 should have been voted on not put forcefully in place that’s not how our government works!!! there has been a decline in youth vaping so now there will be a rise in youth smoking because it’s easy access”
	Inconsistency in adult age definition or limits personal choice	The tweet argues that the policy limits adults’ rights and personal choices.	“yup. not to mention, adults can make their own damn decisions. here in michigan, you now have to be 21 to buy tobacco or vape. i no longer smoke, but if i’m old enough to die for my country, i’m old enough to decide if i buy this shit.”
	Will still use tobacco	The tweet believes that T21 is ineffective in restricting the usage of tobacco among youth, and the underage group will still find a way to purchase tobacco products.	“so, vaping is illegal for &lt;21 in mass and &lt;19 in nh. sounds a lot like alcohol, or cigarettes. kids have been working around these sorts of barriers for generations. vaping regulations are no different than alcohol regulations afa kids are concerned.”
	Not the priority	The tweet argues that T21 should not be the priority of the agenda, and government should focus on policies in other areas.	““the govt acted swiftly when vaping deaths became more frequent. but still stay silent on mass shootings… congress passes bill raising minimum tobacco and vape smoking age to 21.”
	Mocking	The tweet mocks or contains irony and satire about the policy.	“i voted to send the majority leader to washington to repeal and replace obamacare. and all i got was a minimum vaping age. let’s be sure to name a lot buildings and highway after this guy.”
	General disagreement or frustration	The tweet offers no specific reasons for opposing T21 but expresses a negative attitude or frustration in general.	“i knew that also.. and i know i dont like everthing ..like the vape or smoking age to 21 .. but will still vote for him again”
	Other	Other tweets with negative attitudes that do not belong to any of the other categories.	“i almost bought a pack yesterday – i think because it's always in the ether. I'm only a year in so i’'m one of the vulnerable ones – being told by these policies that my health isn't as valuable as these young people's.”
**Neutral**			
	PSA^b^ or news	The content of the tweet is a news headline, which typically states the policy change.	“#us expected to raise #vaping age to 21”
	Dialogue or discussion	The tweet is a part of the conversation or discussion among users with no obvious attitudes.	“sorry to disappoint you! it is illegal for youth to obtain vaping products, t18 or t21 depending on the state‚Äôs law. it was not even targeted to non smokers. it was and always has been meant for smokers!”
	Extend to other regulations	The tweet discusses and compares the age restrictions across smoking, voting, drinking, etc.	“repealing the 26th amendment to raise the voting age back up to 21 and make the draft only apply to those 21 and older. justify by pointing out the drinking age=21, smoking/vape age going up to 21, buying guns/ammo going up to 21 means voting should go up to 21 also.”

^a^T21: Tobacco 21 Amendment.

^b^PSA: public service announcement.

### Data Analysis

To understand the prevalence of T21-related discussion, we examined the temporal trend of daily counts of T21-related posts over the studied period. In addition, we summarized the distribution of the attitude and the specific topics within each attitude group by calculating the frequencies and proportions of tweets in each topic within each attitude group.

### Ethical Considerations

This study only analyzed publicly available data, and the results do not contain any identifiable information and are presented in aggregate.

## Results

### Codebook Development for Thematic Analysis of T21 Tweets

From our data set of 3197 tweets, we determined that 2169 (67.8%) tweets were relevant with regard to T21-related discussion. Of these 2169 tweets, we hand-coded each to their respective attitude and topic based on our defined codebook, which were mutually exclusive groups (Table 1). Among tweets that portrayed a positive attitude, themes identified included (1) avoidance of further regulation, where the tweet claims that T21 should do its job before implementing additional regulations such as an e-cigarette flavor restriction; (2) Enforce T21, where the tweet calls for thorough execution and enforcement of the policy; (3) health benefits, where the tweet states that T21 can reduce the harm to the health of youth; (4) general support tweets offered no specific reasons for supporting T21 but expresses a positive attitude in general; (5) reduced usage, where the tweet states that T21 will be effective to reduce the usage of tobacco among youth; and (6) other tweets that portrayed a positive attitude but did not fit into any of the other categories. Among tweets that showed a negative attitude, themes included (1) parenting or blaming, where the tweet argues that the problem is not tobacco but other factors; (2) inconsistency in adult age definition or limits personal choice, where the tweet argues that the T21 amendment is limiting adult rights and personal choice; (3) will still use tobacco, where the Twitter user believes that the policy will be ineffective in restricting youth use of tobacco and underage youth will still find a way to obtain tobacco products; (4) mocking, where the tweet mocks or contains irony and satires about the policy; (5) general disagreement or frustration, where the tweet offers no specific reasons for opposing T21 but expresses a negative attitude or frustration in general; and (6) other tweets that showed a negative attitude but did not fit into any of the other categories. Finally, tweets that were more neutral in nature contained themes such as (1) PSA or news, where the tweet contained a news headline that stated the policy change; (2) dialogue or discussion, where the tweet was part of a conversation or discussion among users with no obvious attitudes; and (3) extend to other regulations where the tweet discussed and compares age restrictions across different activities (eg, smoking, drinking, gambling, and voting).

### Temporal Trends of Attitudes Toward T21

**Figure 1:**
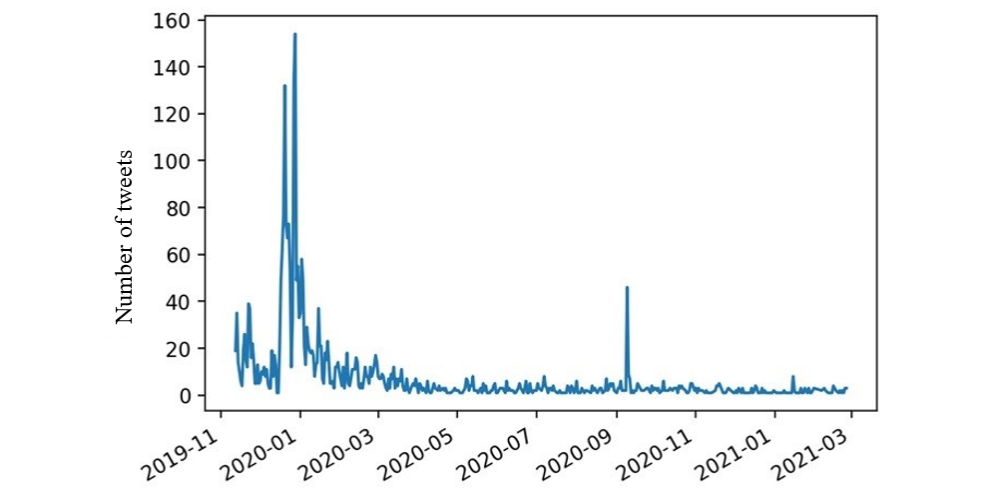
Temporal trend of mentioning Tobacco 21 on Twitter.

It was determined that out of 2169 tweets, 444 (20.47%) showed a positive attitude, 736 (33.93%) showed a negative attitude, and 989 (45.60%) showed a neutral attitude. Figure 1 shows the temporal trend of T21-related mentions on Twitter. There is a clear peak in the number of tweets around January 2020, 2 months following the enactment of this legislation. A secondary peak occurs during September 2020. This secondary peak appears to be associated with a discussion of Florida Governor Ron DeSantis vetoing Florida’s T21 policy and e-cigarette flavor restriction.

### Themes Associated With the Mentioning of T21

The temporal trend of themes associated with the mentioning of T21 was not clearly distinctive from one another and, therefore, is not depicted. Table 2 displays each topic and its associated attitude, amount, and proportion of tweets. Proportions were calculated by attitude. For positive tweets, the most frequent topic was “avoidance of further regulation” (120/444 tweets, 27.0%), followed closely by “Enforce T21” (110/444 tweets, 24.8%), then “health benefit” (81/444 tweets, 18.2%), “other” (78/444, 17.6%), “general support” (34/444 tweets, 7.7%), and “reduce usage” (21/444 tweets, 4.7%). For negative tweets, the most frequent topic was “general disagreement or frustration” (207/736 tweets, 28.1%), followed closely by “will still use tobacco” (188/736 tweets, 25.5%), then “other” (142/736 tweets, 19.3%), “inconsistency in adult age definition or limits personal choice” (70/736 tweets, 15.8%), “mocking” (66/736 tweets, 9.0%), “parenting or blaming” (40/736 tweets, 5.4%), and “not the priority” (23/736 tweets, 3.1%). For neutral tweets, the most frequent topic was “PSA or news” (782/989 tweets, 79.1%), then “dialogue or discussion” (198/989 tweets, 20.0%), and “extended to other regulations” (9/989 tweets, 0.9%).

**Table 2:** Main topics in Tobacco 21–related tweets.

Attitude and topic			N=2169, n (%)
**Positive**			444 (20.5)
	Avoidance of further regulation	120 (27)	
	Enforce T21^a^	110 (24.8)	
	Health benefit	81 (18.2)	
	General support	34 (7.7)	
	Reduce usage	21 (4.7)	
	Other	78 (17.6)	
**Negative**			736 (33.9)
	General disagreement or frustration	207 (28.1)	
	Will still use tobacco	188 (25.5)	
	Inconsistency in adult age definition or limits personal choice	70 (15.8)	
	Mocking	66 (9)	
	Parenting or blaming	40 (5.4)	
	Not the priority	23 (3.1)	
	Other	142 (19.3)	
**Neutral**			989 (45.6)
	PSA^b^ or news	782 (79.1)	
	Dialogue or discussion	198 (20)	
	Extend to other regulations	9 (0.9)	

^a^T21: Tobacco 21 Amendment.

^b^PSA: public service announcement.

## Discussion

### Principal Findings

This analysis used an existing data set of Twitter posts related to vaping and e-cigarettes to assess discussions associated with the signing of T21 in December 2019. Most discussions associated with T21 occurred in the month following the signing of the amendment, with some discussion in the preceding month. A spike in discussions was observed in late 2020. Many tweets at this time discussed Florida Governor Ron DeSantis vetoing Florida’s T21 policy and e-cigarette flavor restriction on September 8, 2020, stating that it was unnecessary due to the Federal T21 policy [36].

The majority of tweets had a neutral attitude toward the T21 amendment. However, more showed a negative attitude than positive. Most negative posts discussed general disagreement and frustration, followed by a discussion that those younger than 21 years will still use tobacco. Studies have assessed the penalty structure for T21 violations and have suggested that monetary fines, no matter the severity, are not as effective as license suspension, revocation, or criminal penalties [37,38]. In addition, local policies with procedures to conduct inspections and impose penalties can ensure the effectiveness and enforcement of T21 [38-40]. Another common topic of discussion was the age definition of an adult; people feel that if one can vote and enlist in the military, then one should be able to purchase tobacco and alcohol. The age of 21 was chosen since most current smokers report trying their first cigarette before turning 21 years old [3]. Furthermore, young adults, 18-25 years of age, are highly influenced by their peers and environment as their brains are still developing [3,41]. However, the Institute of Medicine report on the public health implications of increasing the minimum age to purchase tobacco products found that increasing the age to 25 years as opposed to 21 years would result in considerably smaller effects [42]. Topics of discussion among tweets that showed a positive attitude toward T21 overall had a general theme that enforcement of T21 would work to reduce the use of tobacco and benefit public health. Previous research has also shown the positive impact and great support for increasing the minimum legal age to purchase tobacco to 21 years among various populations and users of tobacco [9,10,12-18,21,40,43].

### Limitations

Social media data can provide rich data on the public’s perception of a policy, such as T21. In addition, the tweets for developing the codebook were randomly sampled and, therefore, can fully represent the whole Twitter data set in our study. However, there are some limitations to this study. First, the demographics of the Twitter users are not available. Furthermore, the geolocation of the users was not available, nor was there a clear distinction if the discussion was referring to the federal policy or a state or local policy, which could be useful to evaluate policies implemented at local and state levels before the federal amendment. Second, less than a quarter of the US population has a Twitter account. In addition, some users have private accounts, so their posts are unavailable. Third, this data set was not collected for the purpose of this analysis, and it focused on vaping and e-cigarettes. Therefore, our findings are only generalizable to users who show an interest in discussing vaping and e-cigarettes on Twitter. Fourth, we only assessed the content of the tweets and no other form of data or interaction between Twitter users (eg, follow, retweet, and favorites). Fifth, there may have been T21 content that was missed due to keyword filtering, and accounts were not assessed to see if they were bots. In addition, about 90% (1952/2169) of tweets in this study were single-coded, which could lead to potential bias even though the intercoder reliability for 10% (217/2169) of tweets was high. Finally, there were a lot of events (eg, COVID-19 and cartridge-based e-cigarette flavor restriction) that may have masked the discussion associated with T21. Therefore, our analysis is not representative of the general population, and an assessment of posts that were not restricted to vaping and e-cigarettes could provide additional information on the public’s perception of the T21 amendment.

### Conclusion

We observed an overall negative public attitude toward the T21 policy on Twitter, with major discussions around the frustration about the T21 policy due to continued underage use of tobacco products. While the attitudes and themes found in this assessment of tweets are consistent with previous studies assessing sentiment toward T21 using Twitter data, this study provided a more comprehensive understanding of reasons either supporting or against T21 policy [32-34]. Greater enforcement and penalties within communities would likely minimize the continued underage use of tobacco products. This analysis of Twitter posts provided a comprehensive look at the public’s perception of the US T21 amendment. Continued monitoring can inform enforcement efforts and potential unintended consequences of T21. Considering more tweets with a negative attitude toward T21 than those with a positive attitude based on our results, it is important to enhance health communication about the underage use of tobacco products, for example, launching health communication campaigns on social media, which can reach more underage population. Furthermore, machine learning models could allow for the assessment of a greater number of tweets or posts found on other platforms that may be lengthier.

## Abbreviations

PSA: public service announcement
T21: Tobacco 21 Amendment

## Data Availability

The data and scripts used for the analysis and creation of figures are available upon request from the corresponding author LMSN.
